# Integrated bioinformatics and machine learning identify early diagnostic biomarkers for MAFLD with comorbid psoriasis

**DOI:** 10.3389/fimmu.2026.1699470

**Published:** 2026-04-15

**Authors:** Shanshan Wang, Yanfang Chen, Tingting Yang, Daming Zhang, Yadi Liu, Shenglan Zhang, Zishan Yang, Tuanjie Wei, Lizhen Qiu, Fang Huang, Zhiqiang Xiao

**Affiliations:** 1 Xinxiang Key Laboratory of Tumor Vaccine and Immunotherapy, School of Basic Medical Sciences, Henan Medical University, Xinxiang, Henan, China; 2 The Biomedical Translational Research Institute, School of Medicine, Jinan University, Guangzhou, China; 3 School of Computer Science, South China Normal University, Guangzhou, China; 4 Center of Mental-Health Education, Jinan University, Guangzhou, China; 5 Department of Dermatology, Zhuhai People’s Hospital (Zhuhai Hospital Affiliated with Jinan University), Jinan University, Zhuhai, China

**Keywords:** biomarkers, immunometabolism, machine learning, MAFLD, psoriasis

## Abstract

**Background:**

Psoriasis and metabolic dysfunction–associated fatty liver disease (MAFLD) share pathological features such as chronic inflammation, immune dysregulation, and metabolic disturbance. Increasing evidence suggests biological crosstalk between the two conditions, offering new insights into their shared mechanisms and comanagement. Early-stage MAFLD, characterized by hepatic steatosis without evident inflammation or fibrosis, provides a crucial window for intervention. This study aimed to identify early diagnostic biomarkers linking psoriasis and MAFLD.

**Methods:**

Transcriptomic datasets of psoriasis and MAFLD were retrieved from the Gene Expression Omnibus (GEO) database. Weighted gene co-expression network analysis (WGCNA) was used to identify MAFLD-related modules. Shared genes were obtained by intersecting module genes with differentially expressed genes (DEGs) from psoriasis datasets. Machine learning algorithms, including random forest (RF), least absolute shrinkage and selection operator (LASSO), and support vector machine (SVM), were applied to identify hub genes. Diagnostic performance was evaluated using receiver operating characteristic (ROC) analysis, immune infiltration assessment, Spearman correlation, and experimental validation in psoriasis and MAFLD mouse models.

**Results:**

Twenty-nine shared genes were identified and found to be enriched in immune and metabolic pathways. Six hub genes—ADRB2, WNT5A, S100A9, FAM110C, S100A12, and TUBB6—were selected through integrated machine learning analysis and experimental validation. These genes exhibited high diagnostic accuracy and significant correlations with disease severity and immune cell infiltration.

**Conclusions:**

This study identified six hub genes—ADRB2, WNT5A, S100A9, FAM110C, S100A12, and TUBB6—as potential cross-disease biomarkers for the comorbidity of psoriasis and MAFLD, and these genes are significantly associated with disease severity. These findings provide new targets for early diagnosis and potential treatment strategies for the comorbidity.

## 1 Introduction

Psoriasis is a chronic immune-mediated inflammatory skin disorder characterized by aberrant activation of the IL-23/Th17 axis (1), (2), genetic predisposition, and environmental triggers such as infections or psychological stress (3), (4). Despite the remarkable efficacy of modern biologic therapies in achieving clinical remission, they fail to completely restore immune homeostasis; Residual pathogenic memory T cells often persist, predisposing patients to disease relapse upon subsequent immune challenges (5), (6). Increasingly, psoriasis is a systemic chronic inflammatory disease whose inflammatory response can extend beyond the skin. Persistent low-grade inflammation establishes a pathological link between skin lesions and various extradermal comorbidities, including cardiovascular disease, metabolic syndrome, and other inflammatory conditions, highlighting the importance of early identification and comprehensive management (7).

Metabolic dysfunction–associated fatty liver disease (MAFLD), formerly termed non-alcoholic fatty liver disease (NAFLD), represents the hepatic manifestation of systemic metabolic dysregulation and has become the most prevalent chronic liver disease worldwide (8), (9). The disease encompasses a pathological continuum ranging from simple steatosis to metabolic dysfunction–associated steatohepatitis (MASH), cirrhosis, and ultimately hepatocellular carcinoma (HCC) (10), (11), (12). Despite its increasing global burden, the pathogenesis of MAFLD remains incompletely elucidated, and effective pharmacological therapies are still lacking (13), (14). The substantial heterogeneity of the disease, along with the intricate crosstalk between immune and metabolic pathways, continues to pose a major obstacle to therapeutic development (15), (16).

The MAFLD spectrum ranges from simple steatosis to metabolic dysfunction–associated steatohepatitis (MASH), cirrhosis, and hepatocellular carcinoma (HCC) (10), (11), (12). Despite its growing burden, the pathogenesis of MAFLD remains incompletely understood, and effective pharmacological treatments remain limited (13), (14). The high heterogeneity of the disease and the intertwined immune and metabolic pathways present a major challenge to drug development (15), (16).

Accumulating evidence highlights a strong epidemiological and mechanistic association between psoriasis and metabolic dysfunction–associated fatty liver disease (MAFLD). Patients with psoriasis exhibit a 1.5- to 3-fold higher prevalence of MAFLD than the general population (17), (18), with reported prevalence rates reaching up to 65% in certain cohorts (18). This comorbidity is thought to stem from shared immunometabolic mechanisms, including chronic systemic inflammation, adipokine dysregulation, and insulin resistance (19), (20). Furthermore, the potential hepatotoxicity of several antipsoriatic therapies underscores the importance of careful assessment of liver function in patients with psoriasis (21), (22).

Recent studies have begun to elucidate the immunological crosstalk and histopathological overlap between psoriasis and MAFLD (23), (24), (25), (26), (27). However, the molecular basis underlying their coexistence remains insufficiently characterized, particularly in the context of early-stage MAFLD, when therapeutic intervention may still halt or reverse disease progression. A deeper understanding of the shared molecular mechanisms could not only facilitate early identification of individuals at high risk for comorbidity but also reveal potential dual-benefit therapeutic targets for both diseases.

In this study, we employed a comprehensive integrative approach that combined weighted gene co-expression network analysis (WGCNA), differential expression analysis, and machine learning algorithms, including RF, LASSO, and SVM, to identify key shared genes between psoriasis and early-stage MAFLD. These hub genes were further validated through receiver operating characteristic (ROC) curve analysis, Spearman correlation analysis, immune cell infiltration profiling, and mouse models of both psoriasis and MAFLD. Collectively, our findings provide novel insights into the shared immunometabolic landscape of psoriasis and MAFLD and may guide the development of early diagnostic biomarkers and targeted therapeutic strategies.

## 2 Materials and methods

### 2.1 Mice

C57BL/6J mice were purchased from the Jackson Laboratory. All mice used in this study were sex- and age-matched and co-housed littermates. All animals were housed in SPF conditions at the Experimental Animal Center of Jinan University. Experiments were conducted using 6 to 8-week-old mice, with all procedures conducted in strict compliance with the ethical regulations and protocols approved by the IACUC of Jinan University.

### 2.2 Data retrieval and preprocessing

The psoriasis-related datasets (GSE13355, GSE30999, GSE114286, GSE121212, GSE85034) and the MAFLD dataset (GSE89632) are from the NCBI GEO database. **Table 1** summarizes the information of psoriasis-related datasets including disease type, platform, sample type, sample size, experimental type, and other reference details.

**Table 1:** Description of GEO datasets analyzed.

Data sets	Platforms	Type of samples	Control sample size	Psoriasis or MAFLD sample size	Applications	References (PMID)
GSE13355 GSE30999 GSE114286 GSE85034	GPL570 GPL570 GPL17303 GPL10558	Psoriasis	64 85 9 30	58 85 18 149	Discovery of DEGs	19169254 22763790 30341238 27667537
GSE89632	GPL14951	MAFLD	24	39	Discovery of Modular genes, Validation of hub genes	35166723 25581263
GSE121212	GPL16791	Psoriasis	27	55	Validation of hub genes	27185339

### 2.3 The data analysis workflow of this study

**Figure 1**.

**Figure 1:**
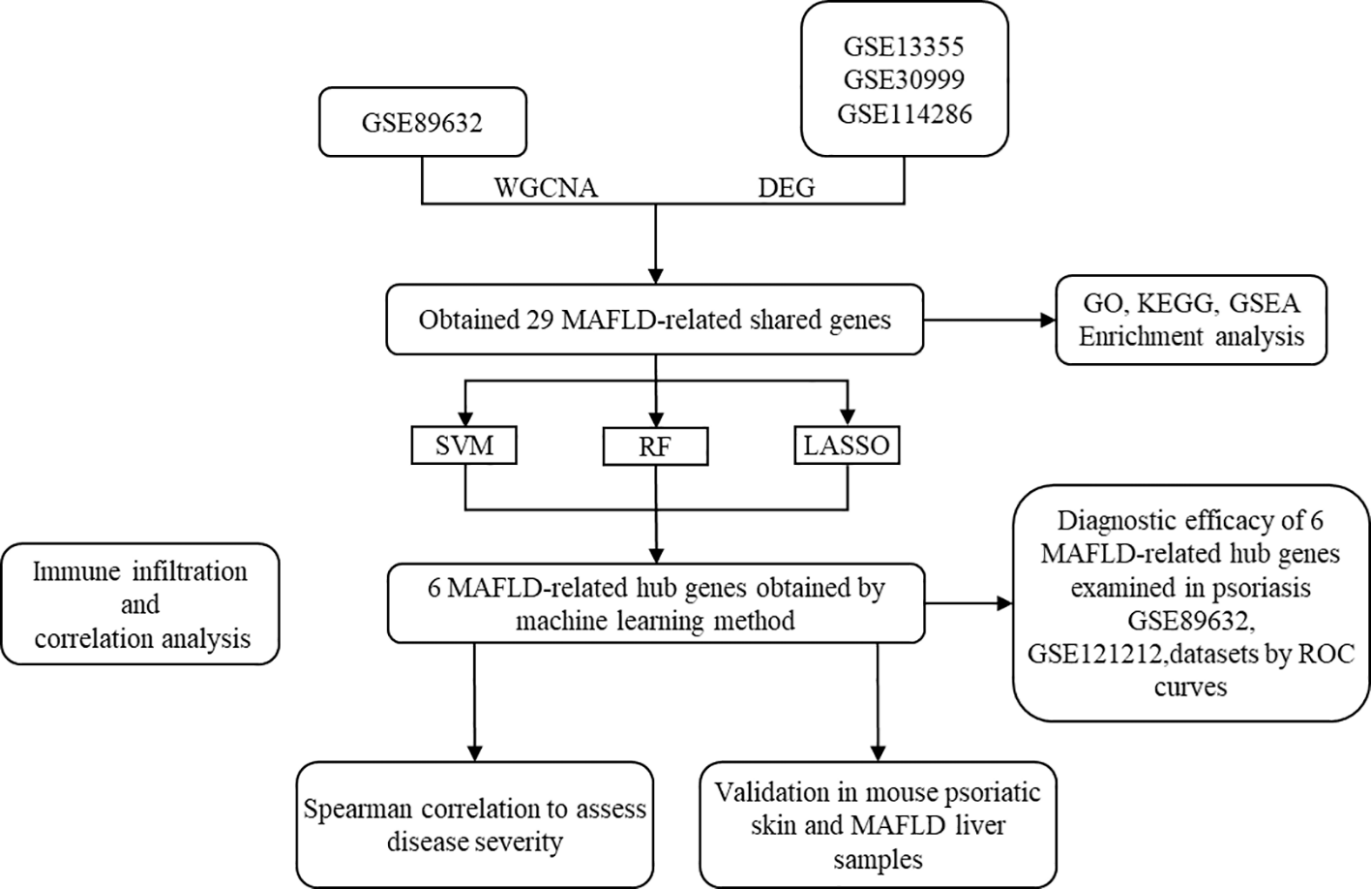
The data analysis workflow of this study.

### 2.4 WGCNA for constructing a co-expression network and identifying module genes

WGCNA is a widely used approach for identifying key genes, co-expression modules, and gene–phenotype associations within biological systems. Since nonalcoholic steatohepatitis (NASH) represents an intermediate stage of MAFLD, NASH samples were excluded to focus on MAFLD-specific gene expression patterns. We selected the GSE89632 dataset to filter MAFLD-related modules and performed WGCNA on all genes using the R package “WGCNA”. Genes were ranked by median absolute deviation (MAD), and the top 5,000 most variable genes were selected for network construction. The scale-free topology of the co-expression network was evaluated under different β shrinkage parameters, and β = 18 was selected based on the scale-free topology criterion. The “dynamic tree cut” method was adopted for module identification, resulting in the successful construction of 7 modules with a merge threshold of 0.25 and a minimum module size of 30. The correlation between modules and sample traits was further analyzed by Pearson correlation tests.

### 2.5 Differential gene expression analysis in psoriasis

Batch effect elimination and quantile normalization were performed on GSE13355, GSE30999, and GSE114286 psoriasis datasets. The threshold for differential expression genes (DEGs) was set to p-value < 0.05 and |log2 FC| ≥ 1.

### 2.6 Identification of shared genes between psoriasis differentially expressed genes and MAFLD-associated modules

The intersection of DEGs from psoriasis and the genes from the blue module of WGCNA in GSE89632 was defined as MAFLD-related shared genes.

### 2.7 KEGG, GO, and GSEA enrichment analysis of MAFLD-related shared genes

The KEGG database is a resource for systematically analyzing the metabolic pathways of gene products within cells and their functional roles, integrating gene and expression data into networks for systematic studies. The Gene Ontology (GO) database helps to understand the common characteristics of genes in a gene set in terms of cellular components (CC), molecular functions (MF), and biological processes (BP). We used the Oebiotech cloud tool to perform KEGG and GO enrichment analyses on MAFLD-related shared genes.

Gene Set Enrichment Analysis (GSEA) is used to evaluate the distribution trend of genes in a pre-defined gene set within a gene expression matrix ranked by phenotype relevance, thereby assessing their contribution to the phenotype. We carried out Gene Set Enrichment Analysis (GSEA) across the whole dataset using the Oebiotech cloud platform to identify significantly enriched pathways and biological processes.

### 2.8 Machine learning-based identification of hub genes

Hub genes were selected using machine learning methods, including Random Forest (RF), Support Vector Machine (SVM), and Least Absolute Shrinkage and Selection Operator (LASSO).

Random Forest (RF) is an ensemble learning method that constructs multiple decision trees during training and outputs the average prediction of the trees, thereby improving model accuracy and reducing overfitting. Least Absolute Shrinkage and Selection Operator (LASSO) performs regression with L1 regularization to penalize the absolute size of coefficients, effectively selecting the most relevant features while preventing overfitting. Support Vector Machine (SVM) identifies an optimal hyperplane that maximizes the margin between classes, making it effective for classification tasks in high-dimensional spaces.

### 2.9 Immune cell infiltration analysis

CIBERSORTx (https://cibersortx.stanford.edu/) is a tool for quantifying immune cell composition from transcriptomic data. Using a support vector machine (SVM) algorithm and a gene expression matrix of immune cell markers, it estimates the relative proportions of immune cell types. We applied CIBERSORTx to the psoriasis datasets to assess the composition and abundance of 22 immune cell types and explore the correlation between MAFLD-related hub gene expression and immune cell infiltration.

### 2.10 Induction of the mouse psoriasis model

The inducing agent used in this experiment was imiquimod (IMQ) manufactured by Sichuan Mingxin Lidai Co., Ltd. Each box contains 4 packets, each containing 250 mg IMQ. An 8-week-old mouse model of psoriasis was established. Mice were randomly divided into groups of 6. After removing hair from the backs of the mice, IMQ was applied topically daily for 7 consecutive days at a dose of 62.5 mg/day until the psoriatic lesions reached their peak. Subsequently, the mice were anesthetized by intraperitoneal injection and sacrificed by cervical dislocation. Skin samples were collected from the backs of the mice for qPCR analysis. The Psoriasis Area Severity Index (PASI) scoring criteria were used. Erythema, scaling, and thickening were scored independently, ranging from 0 to 4 points (0 = none; 1 = mild; 2 = moderate; 3 = significant; 4 = very significant). The cumulative score (erythema + scaling + thickening) for each mouse ranged from 0 to 12 points.

Anesthetic Preparation: To prepare 100 ml of anesthetic, 1.25% tribromoethanol is required. Dissolve 1.25 g of tribromoethanol (T48402–25 g, Sigma) and 2.5 ml of tert-amyl alcohol (152463–250 ml, Sigma) in 97.5 ml of double-distilled water. Stir overnight at room temperature, protected from light. Store at 4 degrees Celsius for up to one month.

### 2.11 RNA isolation and quantitative real-time PCR

Total RNA was isolated from frozen tissue samples using the TRNzol Universal Reagent (TianGen, China) following the manufacturer’s protocol. RNA purity and concentration were assessed using a NanoDrop 2000 UV-Vis spectrophotometer (Thermo Fisher Scientific, USA). Primer sequences used for subsequent analyses are listed in **Supplementary Table 1**.

### 2.12 ROC curve to assess diagnostic value

The Receiver Operating Characteristic (ROC) curve is an important tool for evaluating the performance of classification models, visualizing the relationship between the true positive rate (TPR) and false positive rate (FPR) at various threshold settings. ROC curves were generated to assess the diagnostic performance of the hub genes identified in this study, providing insights into their potential for distinguishing between different phenotypic groups.

### 2.13 Verification of MAFLD-related hub genes expression in the psoriasis data sets

The GSE121212 psoriasis dataset was used to validate the expression levels of NAFLD-related hub genes.

### 2.14 Histology and immunostaining

Mouse back skin tissue samples were fixed with 4% paraformaldehyde, embedded in paraffin, and sliced before hematoxylin-eosin (H&E) staining. All technical services were provided by Wuhan Saiweier Biotechnology Co., Ltd.

### 2.15 Induction of MAFLD model

To establish the MAFLD model, 8-week-old male mice were fed a high-fat diet (HFD; 60% kcal from fat; D12492, Research Diets) for 10 consecutive weeks. Body weight was recorded weekly throughout the feeding period. At the end of the experiment, mice were euthanized, and liver tissues were harvested for histological analysis. Hematoxylin and eosin (H&E) staining was performed to assess hepatic steatosis and inflammation, and Oil Red O staining was conducted on frozen sections to evaluate lipid accumulation.

### 2.16 Data statistics and analysis

All experiments were conducted in triplicate to ensure reproducibility, with consistent results observed across repetitions. GraphPad Prism (v.8) was utilized for data visualization and statistical analysis. Detailed descriptions of statistical tests are provided in the figure legends, with all data conforming to normal distribution and similar variance. Comparisons between two groups were made using a two-tailed Student’s t-test. Statistical significance in the experimental results is indicated as follows: * represents p < 0.05, ** represents p < 0.01, *** represents p < 0.001, and ns indicates no significant difference.

## 3 Results

### 3.1 WGCNA identifies key modules of MAFLD-related genes

**Figure 2:**
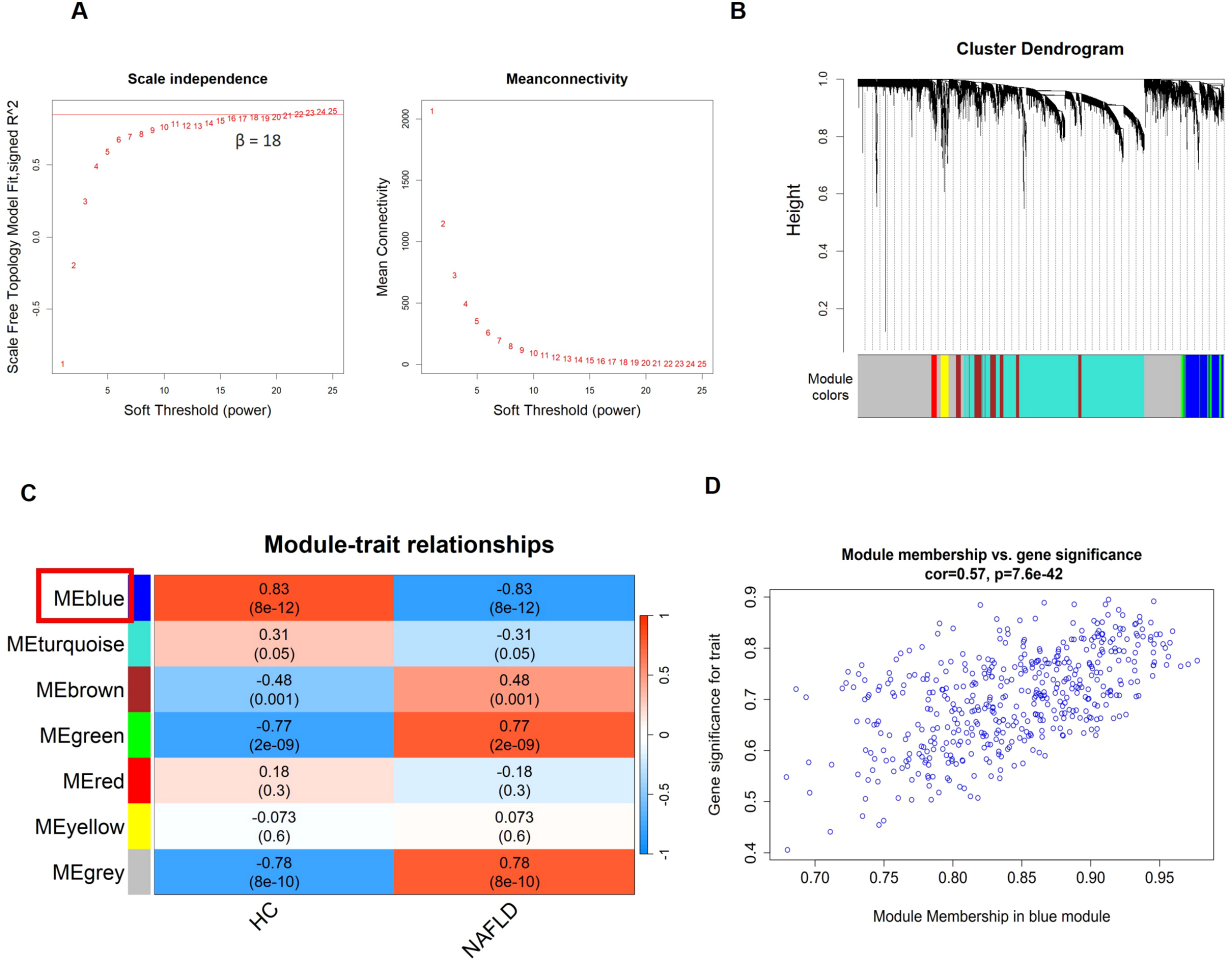
WGCNA identifies key modules of MAFLD-related genes. **(A)** Select β = 18 as the soft-thresholding parameter to construct the scale-free network. **(B)** Determine 7 co-expression modules using hierarchical clustering based on dynamic tree cut. **(C)** Heatmap analysis of the correlation between module eigengenes and clinical traits. **(D)** Scatter plot of the correlation between the blue module and MAFLD genes.

WGCNA was conducted using the GSE89632 dataset to identify gene modules most correlated with MAFLD. A soft-thresholding power of β = 18 (scale-free R² = 0.85) was selected to construct a scale-free network **Figure 2**). Subsequently, dynamic tree cutting was applied to identify seven gene co-expression modules (GCMs) (**Figure 3.1**). To evaluate the correlation between modules and MAFLD, Pearson’s correlation coefficients were calculated between module eigengenes and MAFLD (**Figure 2C**, **Supplementary Table 2**). The blue module showed the strongest positive correlation with MAFLD (R² = 0.83, p = 8 × 10^-12^), indicating its potential role in MAFLD progression.

### 3.2 Identification of shared genes between psoriasis and MAFLD

**Figure 3:**
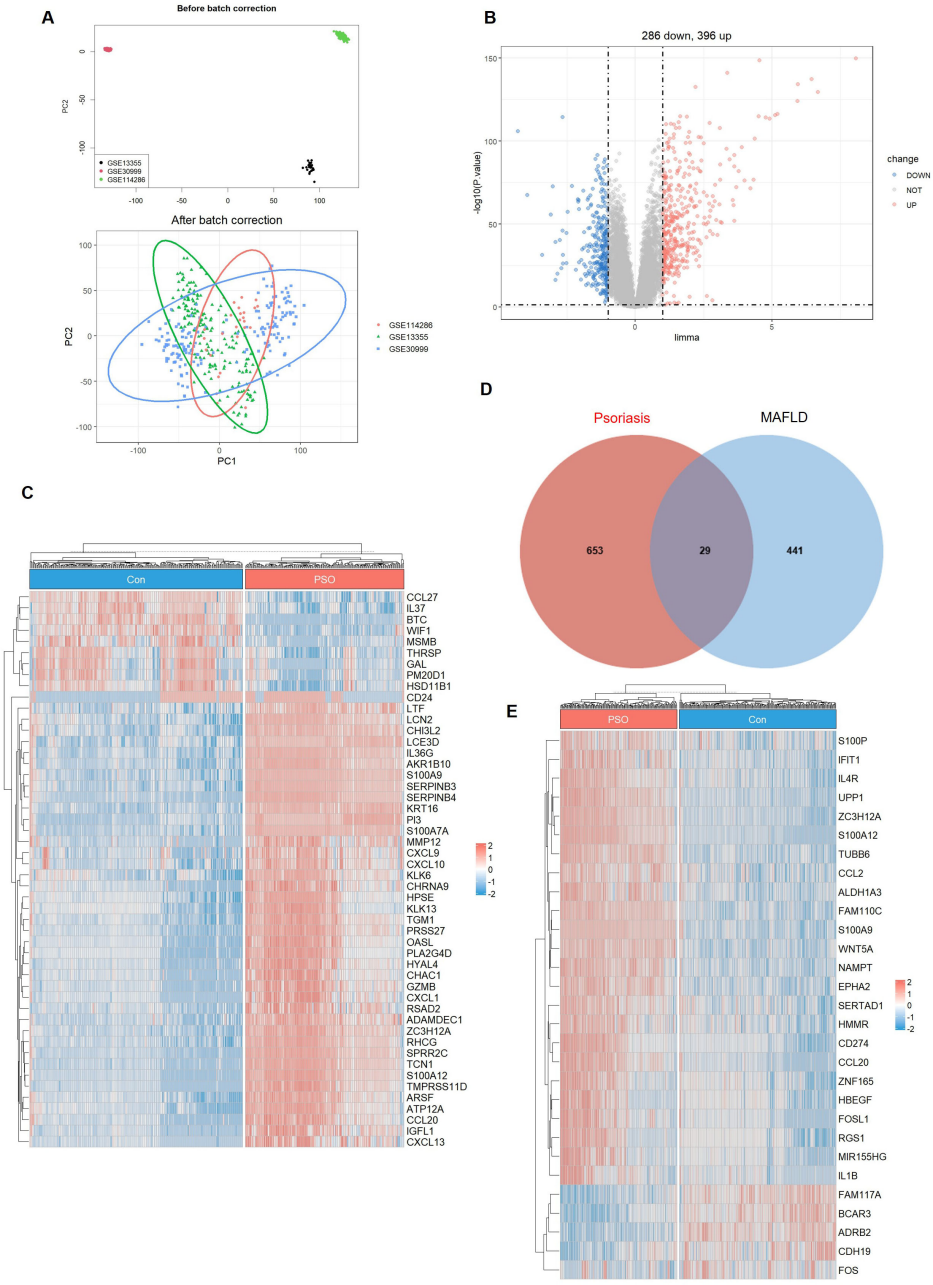
Identification of shared genes between psoriasis and MAFLD. **(A)** Batch Effect Correction for Psoriasis Datasets. **(B)** Volcano plot of DEGs in the three Psoriasis datasets. **(C)** Heatmap of the top 50 most significantly differentially expressed genes. **(D)** Intersection of genes from the blue module in WGCNA and differentially expressed genes (DEGs) in the Psoriasis datasets. **(E)** Heatmap of the 29 shared genes.

We obtained the GSE13355, GSE30999, and GSE114286 psoriasis datasets from the GEO database and removed the batch effect of the datasets (**Figure 3**). We performed differentially expressed gene (DEG) analysis on the psoriasis datasets. We identified 396 upregulated and 286 downregulated genes (**Figure 3**, **Supplementary Table 3**). A heatmap of the top 50 DEGs is shown in **Figure 3**. Intersection analysis of psoriasis-associated DEGs with the 470 genes within the MAFLD-linked blue module identified 29 overlapping genes functionally relevant to MAFLD, which were graphically represented using Venn diagram and heatmap. (**Figure 3D**, **Supplementary Table 4**).

### 3.3 Enrichment analysis of MAFLD-related shared genes

**Figure 4:**
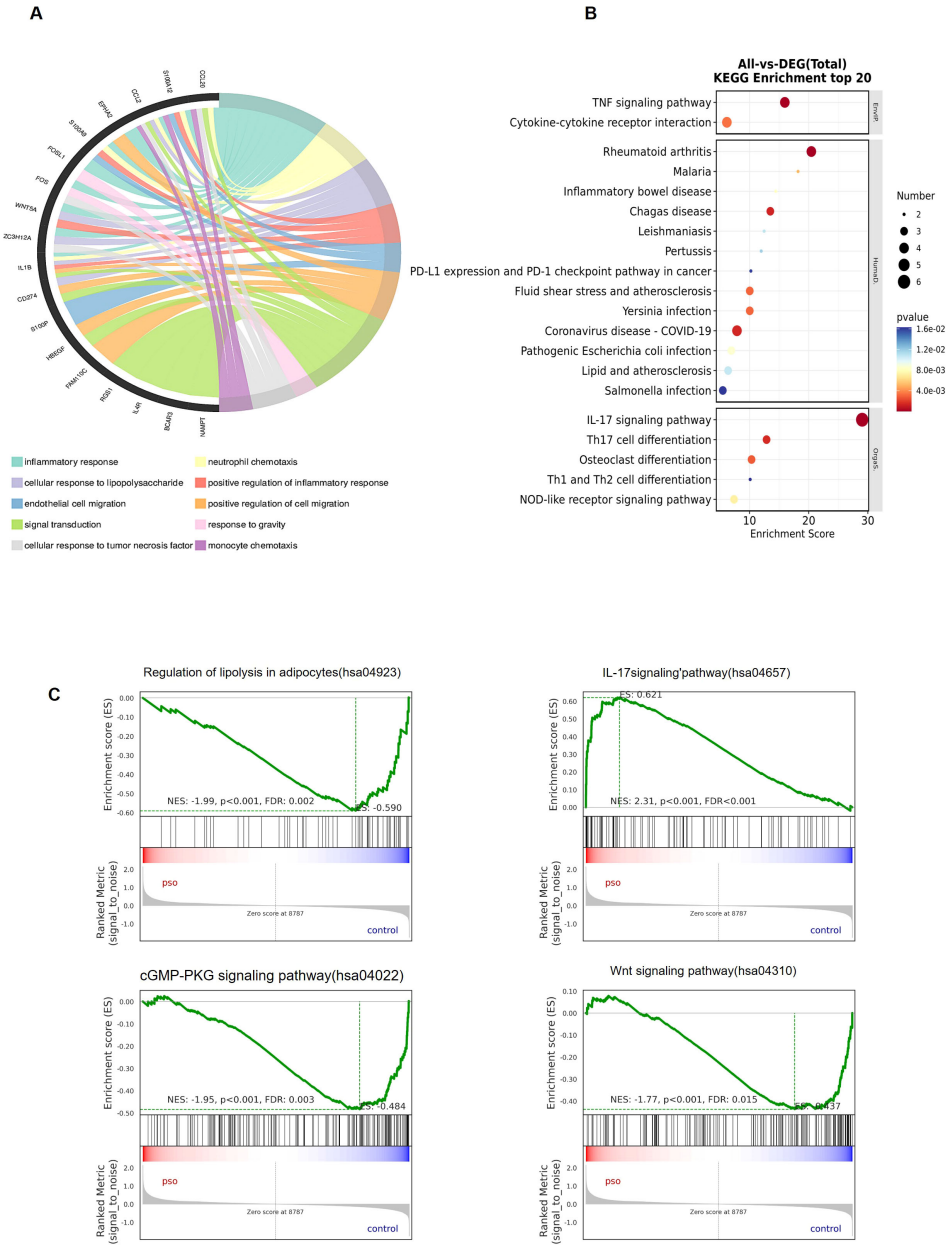
Enrichment analysis of MAFLD-related shared genes. **(A-C)** GO, KEGG, and GSEA enrichment analysis of the 29 MAFLD-related shared genes.

To explore the mechanistic roles of MAFLD-associated shared genes in psoriasis progression, GO, KEGG, and GSEA were performed on the 29 shared genes. GO chord top analysis showed that these 29 genes were primarily associated with immune-related pathways, including inflammatory response, neutrophil chemotaxis, and signal transduction (adjusted p-value < 0.05; **Figure 4**). KEGG pathway analysis revealed significant enrichment in TNF signaling, rheumatoid arthritis, IL-17 signaling, Lipid and atherosclerosis (**Figure 4**). GSEA further highlighted enriched pathways such as Regulation of lipolysis in adipocytes, Wnt signaling pathway, IL-17 signaling and cGMP-PKG signaling pathway (**Figure 4**). Collectively, these findings suggest that the 29 MAFLD-related shared genes play a pivotal role in orchestrating systemic metabolic-immune crosstalk, potentially bridging hepatic steatosis and psoriatic inflammation.

### 3.4 Machine learning-based identification of hub genes

**Figure 5:**
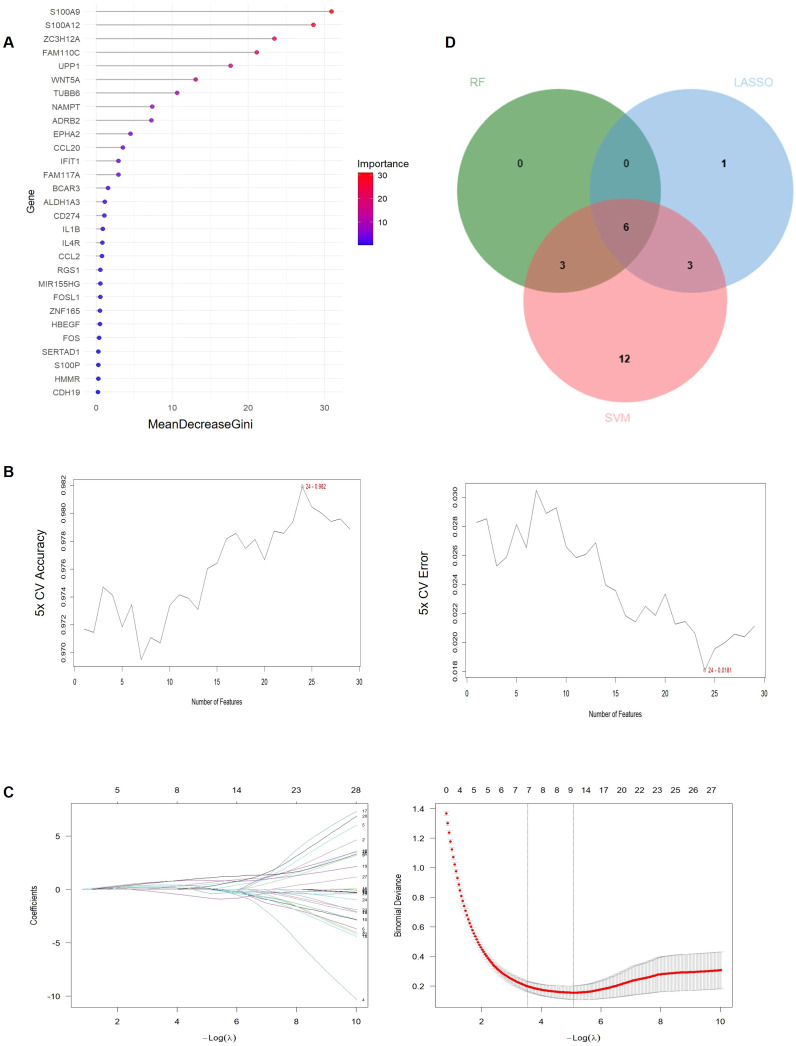
Machine learning-based identification of hub genes. **(A)** Random forest analysis of 29 MAFLD-related hub genes. **(B)** SVM analysis of 29 MAFLD-related hub genes. **(C)** LASSO analysis of 29 MAFLD-related hub genes. **(D)** Venn diagram of the intersection between Random Forest, SVM, and LASSO.

To identify hub genes associated with MAFLD, we applied machine learning algorithms to the 29 shared genes. Random Forest identified 9 hub genes with MeanDecreaseGini > 5 (**Figure 5**). LASSO identified 10 hub genes (**Figure 3.4**), and Support Vector Machine (SVM) identified 24 hub genes (**Figure 3.4**). The intersection of results from all three algorithms yielded six hub genes: *ADRB2*, *WNT5A*, *S100A9*, *FAM110C, S100A12, and TUBB6* (**Figure 5**, **Supplementary Table 5**).

### 3.5 Assessing the diagnostic value and clinical relevance of hub genes

**Figure 6:**
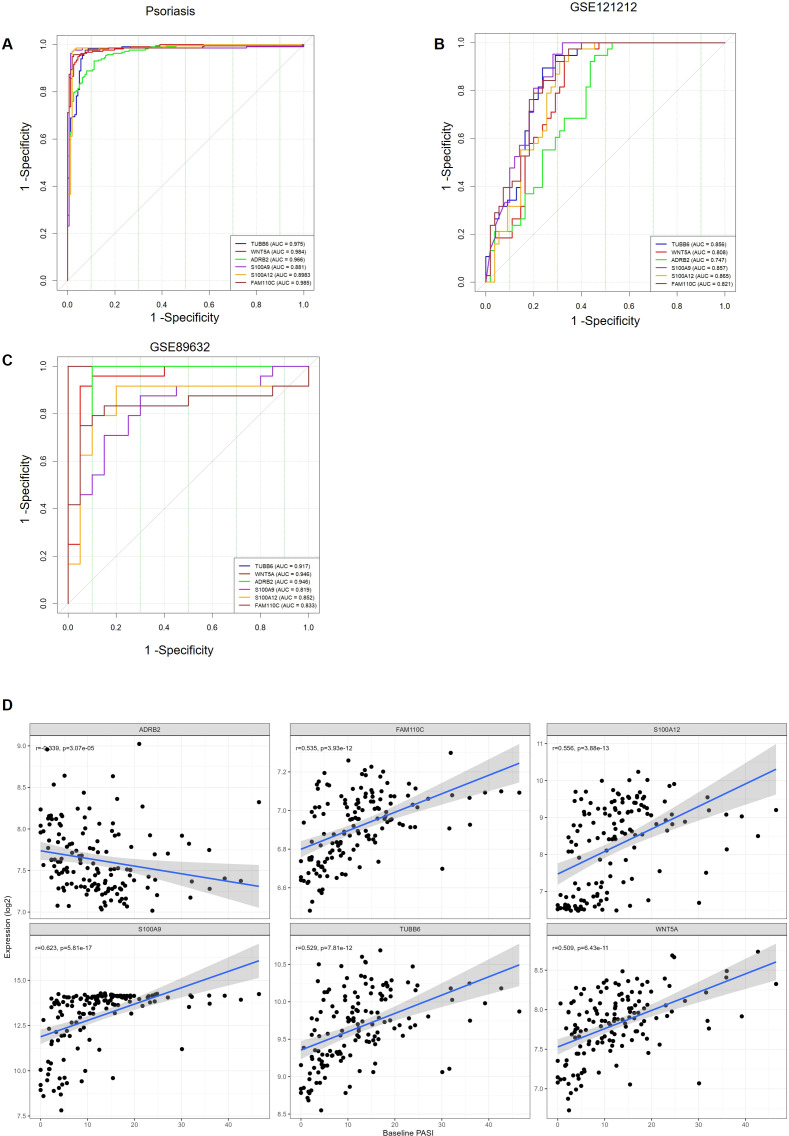
Diagnostic value of hub genes assessed by ROC curves. **(A–C)** The areas under the ROC curves (AUC) of 6 MAFLD-related hub genes in the datasets psoriasis, GSE121212, GSE89632. **(D)** Spearman correlation to assess the disease severity of 6 MAFLD-related hub genes.

To evaluate the diagnostic performance of the six hub genes, we generated ROC curves in the psoriasis dataset. The areas under the ROC curves were 0.966, 0.984, 0.881, 0.985,0.8983, and 0.975 for *ADRB2, WNT5A, S100A9, FAM110C, S100A12, and TUBB6*, respectively (**Figure 2**). Furthermore, we validated their diagnostic value in the GSE121212, and GSE89632 datasets, and the results were nearly consistent (**Figures 3.5B**). In the GSE85034 dataset, we used Spearman correlation analysis to assess the association between the expression levels of six key genes and psoriasis severity (measured by the Psoriasis Area and Severity Index [PASI]) (**Figure 6**). The results showed that ADRB2 expression was significantly negatively correlated with PASI, while the expression levels of WNT5A, S100A9, FAM110C, S100A12, and TUBB6 were significantly positively correlated with PASI. These findings indicate that these six hub genes are not only strongly associated with disease severity but also exhibit substantial discriminatory power in patient stratification and disease assessment, underscoring their potential clinical translational value.

### 3.6 Validation of hub genes in a psoriasis mouse model and MAFLD mouse model

**Figure 7:**
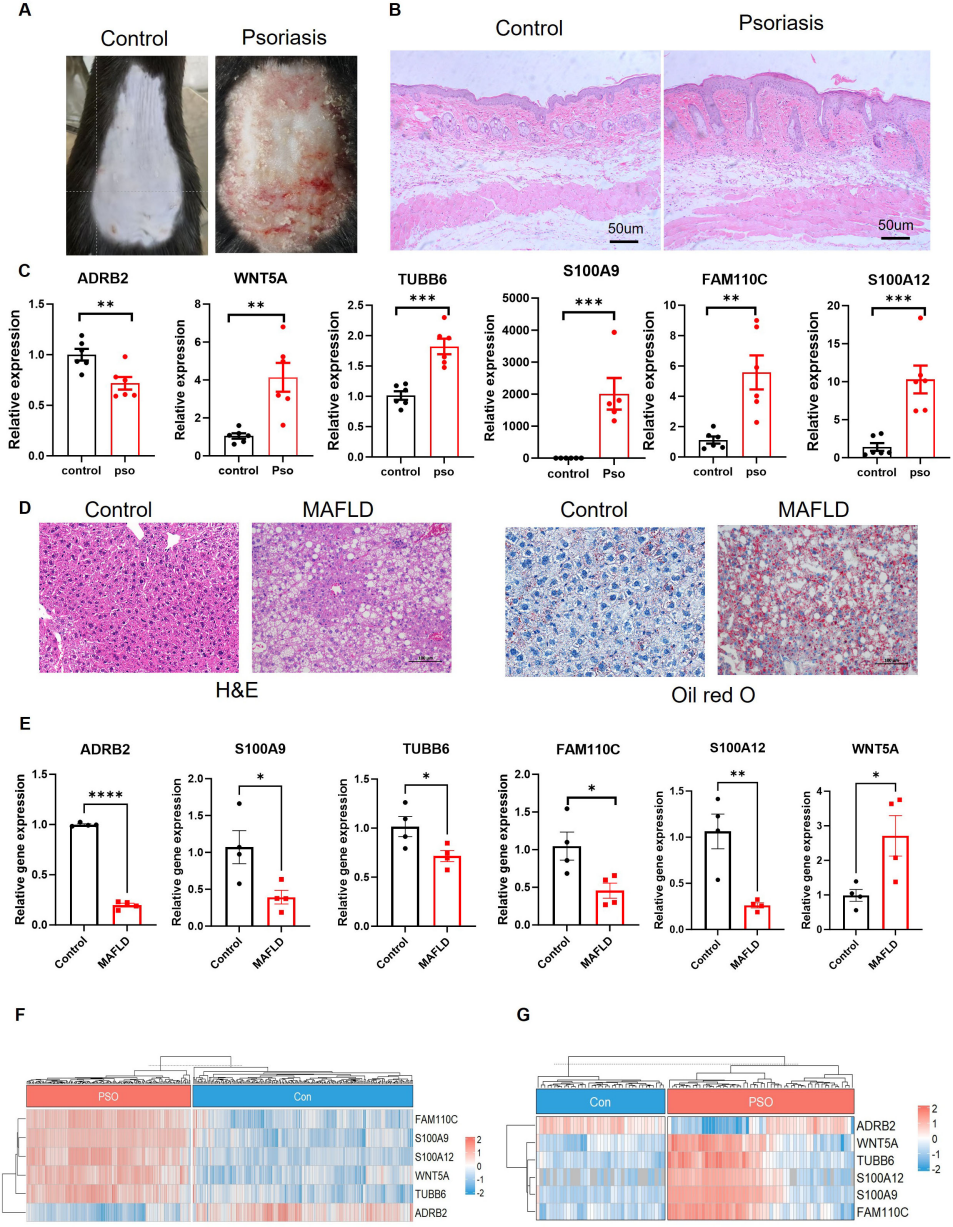
Validation of hub genes in a psoriasis mouse model and MAFLD mouse model. **(A)** Representative images of the back skin of mice after IMQ-induced psoriasis. **(B)** Representative images of H&E-stained sections of psoriasis and control mouse skin. Scale bar, 50 um. **(C)** qPCR analysis of the expression levels of 6 hub genes in psoriasis mouse skin tissue. **(D)** Hematoxylin and eosin (H&E) staining and Oil Red O staining of Liver tissues. Scale bars, 100 µm. **(E)** qPCR analysis of the expression levels of 6 hub genes in MAFLD mouse liver tissue. **(F–G)** Heatmap visualization of the expression profiles of six key genes in the psoriasis training dataset (left) and the validation dataset GSE121212 (right), illustrating the consistency of expression patterns across datasets. The symbols denote statistical significance: * p < 0.05, ** p < 0.01, *** p < 0.001, and **** p < 0.0001.

To validate the expression of candidate genes in psoriasis, we used an imiquimod (IMQ)-induced psoriasis mouse model. After IMQ induction, the back skin of mice showed obvious psoriasis, redness, swelling and thickening (**Figure 7A**). Reverse Transcription Quantitative PCR (RT-qPCR) analysis revealed significantly upregulated mRNA expression levels of six hub genes—*ADRB2, WNT5A, S100A9, FAM110C, S100A12, and TUBB6*—in lesional skin tissues of psoriasis model mice compared to control mice (**Figure 7**). Meanwhile, to verify that the hub genes we identified exert similar effects in the MAFLD model, we induced fatty liver disease in mice. Hematoxylin–eosin (H&E) and Oil Red O staining confirmed the successful establishment of the MAFLD model (**Figure 7**). Reverse transcription quantitative PCR (RT-qPCR) analysis revealed that the hepatic tissues of MAFLD mice exhibited significantly increased mRNA expression levels of the six hub genes — ADRB2, WNT5A, S100A9, FAM110C, S100A12, and TUBB6 (**Figure 7**). These findings demonstrate cross-species conservation of these genes in the pathogenesis of both diseases and further suggest their potential as biomarkers for the development of psoriasis in MAFLD patients. In addition, in the GSE121212 psoriasis dataset (**Figure 3.6**), six hub genes showed significant differences, which was consistent with the changing trend of our mouse psoriasis model, further indicating that the six NAFLD-related genes can be used as effective markers for the diagnosis of psoriasis.

### 3.7 Immune cell infiltration analysis

**Figure 8:**
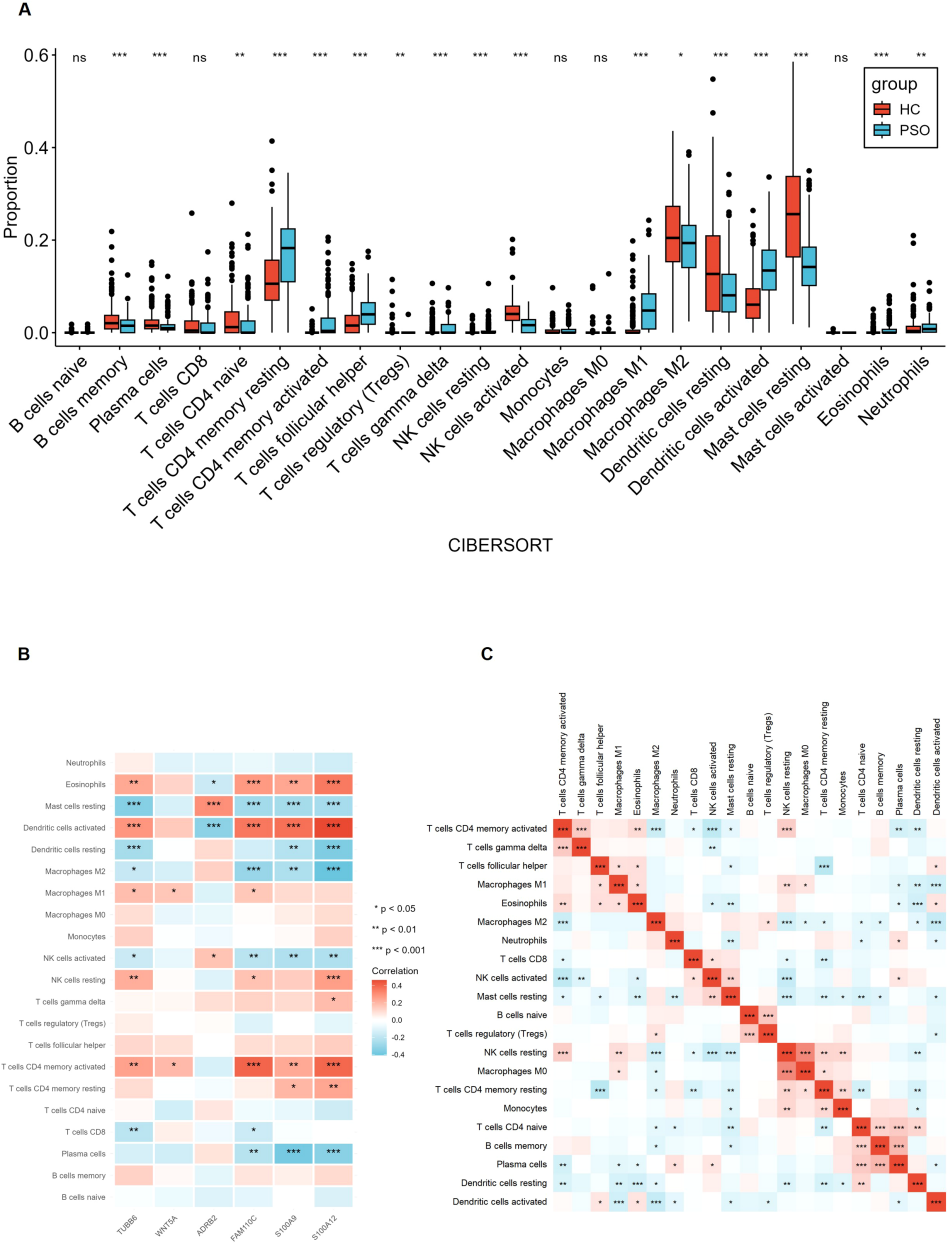
Immune cell infiltration analysis. **(A)** CIBERSORTx analysis of immune cell infiltration in psoriasis. Histogram of 22 immune cell types. **(B)** Heatmap analysis of the correlation between 22 immune cell types and 6 key genes. **(C)** Heatmap analysis of the correlation between 22 immune cell types. The symbols denote statistical significance: * p < 0.05, ** p < 0.01, *** p < 0.001, and **** p < 0.0001.

Our previous GO and KEGG enrichment analyses revealed significant enrichment of immune-related signaling pathways, prompting the hypothesis that immune cells may contribute to the pathogenesis of psoriasis. To further investigate this, we analyzed the correlation between six hub genes (ADRB2, WNT5A, S100A9, FAM110C, S100A12, and TUBB6) and each of the immune cell types in psoriatic lesions. Subsequent analysis using CIBERSORTx, a transcriptome-based tool for immune cell quantification, confirmed that psoriasis development disrupts the skin immune microenvironment, with significant alterations in the composition of locally infiltrating immune cell subsets (**Figure 8**). Correlation analysis between the hub genes and immune cell infiltration demonstrated distinct expression patterns (**Figure 8**): ADRB2 demonstrated significant upregulation in Mast cells resting but downregulation in Dendritic cells activated. S100A9 was markedly upregulated in Dendritic cells activated but downregulated in Activated memory CD4⁺ T cells. FAM110C exhibited upregulation in T cells CD4 memory activated, Dendritic cells activated, and Eosinophils, whereas downregulation was observed in Macrophages M2, and Mast cells resting. WNT5A showed increased expression in Macrophages M1 but decreased expression in Activated memory CD4⁺ T cells. S100A12 exhibited upregulation in T cells CD4 memory activated, Dendritic cells activated, and Eosinophils, whereas downregulation was observed in Dendritic cells resting, Macrophages M2, and Plasma cells. TUBB6 demonstrated significant upregulation in Dendritic cells activated but downregulation in Mast cells resting, Dendritic cells activated.

In addition, NK cells and M0 macrophages showed the strongest positive correlation with each other (r = 0.65). T cells, CD4 memory activated, and NK cells activated exhibited the strongest negative correlation (r = -0.36) (**Figure 8**). Notably, the six hub genes displayed divergent correlation trends with immune cell infiltration, suggesting their potential involvement in regulating immune cell recruitment through distinct molecular mechanisms. These findings highlight the multifaceted roles of NFKBIZ, S100A9, UPP1, and WNT5A in shaping the immune landscape of psoriatic lesions.

## 4 Discussion

Metabolic dysfunction-associated fatty liver disease (MAFLD) is a highly prevalent chronic liver condition, affecting approximately 25% of the global population, particularly among individuals with metabolic syndromes such as obesity, type 2 diabetes, and hypertension (7). The disease spectrum of MAFLD ranges from simple hepatic steatosis to more severe manifestations including inflammation, fibrosis, cirrhosis, and hepatocellular carcinoma, posing a substantial public health burden (7). Importantly, MAFLD is strongly associated with chronic low-grade inflammation and immune dysregulation—features it shares with psoriasis, a systemic inflammatory skin disease. Both conditions are driven by overlapping pathological pathways, such as the IL-23/Th17 and NF-κB axes. Although previous studies have explored the epidemiological link between MAFLD and psoriasis, the molecular mechanisms underpinning their co-occurrence remain largely unexplored. Thus, elucidating the molecular association between psoriasis and early MAFLD holds great significance for improving early diagnosis and therapeutic strategies for both diseases.

In this study, we integrated bioinformatics and machine learning approaches to identify six hub genes—ADRB2, WNT5A, S100A9, FAM110C, S100A12, and TUBB6—as potential cross-disease biomarkers. These genes were consistently validated in external datasets and mouse models of psoriasis and MAFLD, suggesting that they may play an important role in mediating the immunometabolic crosstalk between psoriasis and MAFLD.

ADRB2, a β2-adrenergic receptor, serves as a critical regulator at the intersection of immune responses and metabolic homeostasis. In psoriasis, ADRB2 activation influences immune cell function by modulating cytokine secretion, T cell polarization, and neutrophil activity, thereby amplifying cutaneous inflammation (28). Dysregulated ADRB2 signaling has been associated with enhanced production of pro-inflammatory mediators, including IL-17 and IL-23 axis components, which are central to psoriatic pathogenesis. In the context of MAFLD, Aberrant ADRB2 signaling contributes to chronic low-grade inflammation and metabolic dysfunction, thereby promoting hepatic steatosis and progression toward steatohepatitis (29). Importantly, ADRB2-mediated immunometabolic pathways may represent a shared pathogenic mechanism linking psoriasis and MAFLD.

WNT5A, a non-canonical Wnt signaling ligand, is markedly overexpressed in psoriatic skin lesions and contributes to epidermal thickening and immune cell activation through Ca²⁺/NFAT and JNK signaling pathways. In MAFLD, WNT5A aggravates hepatic insulin resistance and inflammation (30). Its dual role in both skin and liver inflammation positions WNT5A as a promising target for combined intervention.

FAM110C belongs to the FAM110 protein family and is primarily localized in the cytoplasm and microtubule-organizing centers, playing a role in cell cycle regulation, cell migration, and immune signaling. Previous studies have suggested that FAM110C interacts with signaling molecules such as AKT1, potentially affecting immune cell proliferation and inflammatory responses (31). In MAFLD, low FAM110C expression is associated with impaired lipid metabolism and increased inflammatory cell infiltration, suggesting its involvement in maintaining liver immune-metabolic homeostasis. Although direct mechanistic experimental evidence is currently lacking, combined with its functional clues in cell cycle and immune metabolism, FAM110C may serve as a common immune-metabolic regulator in both psoriasis and MAFLD, worthy of further study and clinical validation.

S100A12 and S100A9 are calcium-binding proteins of the S100 family that function as damage-associated molecular patterns (DAMPs), amplifying inflammation through interaction with receptors such as RAGE and TLR4. In psoriasis, both proteins are highly upregulated in keratinocytes and infiltrating immune cells, driving neutrophil chemotaxis, IL-17/IL-23 axis activation, and sustained cutaneous inflammation (32). In MAFLD, S100A12 and S100A9 contribute to hepatic immune-metabolic imbalance by promoting macrophage recruitment, oxidative stress, and hepatocyte injury, thereby accelerating the progression from steatosis to steatohepatitis. Importantly, their dual role in inflammation and metabolism positions S100A12 and S100A9 as shared pathogenic mediators that link systemic inflammation in psoriasis with metabolic dysfunction in MAFLD (33). These proteins thus represent promising biomarkers and potential therapeutic targets for the management of both diseases.

TUBB6 encodes a β-tubulin isoform involved in cytoskeletal organization, cell proliferation, and immune cell motility. In psoriasis, dysregulated TUBB6 expression has been linked to aberrant keratinocyte proliferation and altered immune cell dynamics within skin lesions, suggesting a role in sustaining chronic inflammation (34). In MAFLD, TUBB6 contributes to hepatocyte cytoskeletal remodeling and lipid droplet dynamics, potentially influencing hepatic lipid accumulation and inflammatory cell infiltration (35). Collectively, TUBB6 may act as a shared cytoskeletal and immunometabolic regulator in psoriasis and MAFLD, with potential implications as a biomarker and therapeutic target.

Based on our findings, we hypothesize that these six hub genes constitute an immunometabolism network that underlies the comorbidity between psoriasis and MAFLD. ADRB2 regulates immune cell activity and energy metabolism through the β-adrenergic receptor/cAMP-PKA signaling pathway, linking chronic inflammation to systemic metabolic dysfunction. WNT5A activates the non-canonical Wnt/PCP and Wnt/Ca²⁺ pathways, playing a central role in regulating keratinocyte proliferation, immune cell migration, and hepatic lipid metabolism. S100A9 and S100A12 act as damage-associated molecular patterns (DAMPs), amplifying inflammatory responses through the RAGE/TLR4-NF-κB cascade, promoting neutrophil recruitment, proinflammatory cytokine release, and disrupting hepatic immune-metabolic homeostasis. Although research on FAM110C is limited, it is hypothesized to be involved in cell cycle regulation pathways (such as p53 and PI3K-AKT), potentially playing a protective role in maintaining immune balance and metabolic homeostasis. TUBB6, a cytoskeletal protein, influences microtubule dynamics, thereby regulating keratinocyte proliferation, immune cell motility, and hepatocyte lipid droplet transport and metabolism. Overall, these genes interact through key immune-metabolism signaling networks, including β-adrenergic signaling, the Wnt non-canonical pathway, RAGE/TLR4-NF-κB, JAK-STAT, and PI3K-AKT, to drive chronic inflammation and metabolic imbalance. This suggests a central role in the pathogenesis of psoriasis and MAFLD, providing potential avenues for targeted therapy.

Nevertheless, this study has several limitations. First, our analyses relied on publicly available transcriptomic datasets, which may not fully capture protein-level changes or post-translational modifications. Second, although key findings were partially validated in a psoriasis mouse model, no single-cell RNA sequencing was performed; therefore, the cellular heterogeneity and precise cell-type–specific effects of the identified genes remain unclear. Third, potential data heterogeneity and residual batch effects in the transcriptomic datasets, as well as differences between the mouse model and human disease, may limit the generalizability of our findings. Finally, the lack of protein-level or functional validation further constrains the mechanistic interpretation. Future studies integrating multi-omics approaches (e.g., proteomics, metabolomics, and single-cell transcriptomics) and well-characterized clinical cohorts are essential to elucidate causal mechanisms and enhance the translational relevance of these results.

## 5 Conclusion

This study identified six immune metabolic hub genes—ADRB2, WNT5A, S100A9, FAM110C, S100A12, and TUBB6—as potential early diagnostic biomarkers for the comorbidity of psoriasis and multiple fatty liver disease (MAFLD), significantly associated with disease severity. These findings provide a theoretical basis for developing combined skin and liver diagnostic tools for patients with metabolic-inflammatory syndromes and exploring targeted treatment strategies.

## Data Availability

The original contributions presented in the study are included in the article/**Supplementary Material**. Further inquiries can be directed to the corresponding authors.
