# Supplementary material for: Integrated bioinformatics and machine learning identify early diagnostic biomarkers for MAFLD with comorbid psoriasis
Source: Front Immunol. 2026 Apr 15;17:1699470. doi: 10.3389/fimmu.2026.1699470 (PMC13125174; doi:10.3389/fimmu.2026.1699470)
Supplement: Supplementary file 1 [file DataSheet1.zip › Supplementary material/code.zip!/code/code/WGCNA/3.power/step2_power-value.pdf]

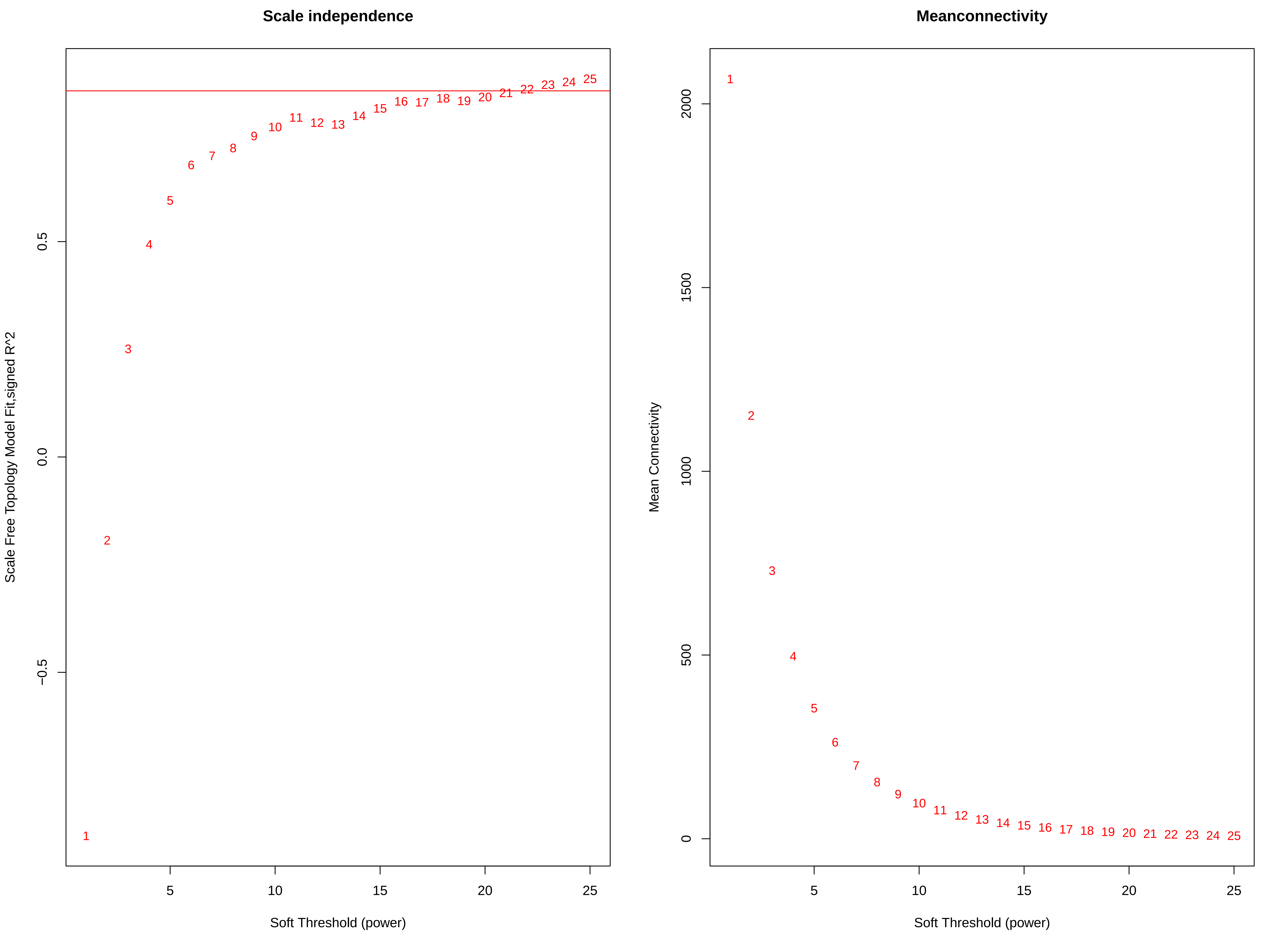

Scale independence
Scale Free Topology Model Fit,signed R^2
Soft Threshold (power)
Meanconnectivity
Mean Connectivity
Soft Threshold (power)
