# Supplementary material for: Integrated bioinformatics and machine learning identify early diagnostic biomarkers for MAFLD with comorbid psoriasis
Source: Front Immunol. 2026 Apr 15;17:1699470. doi: 10.3389/fimmu.2026.1699470 (PMC13125174; doi:10.3389/fimmu.2026.1699470)
Supplement: Supplementary file 1 [file DataSheet1.zip › Supplementary material/code.zip!/code/code/WGCNA/4.one step/step4_genes-modules_ClusterDendrogram.pdf]

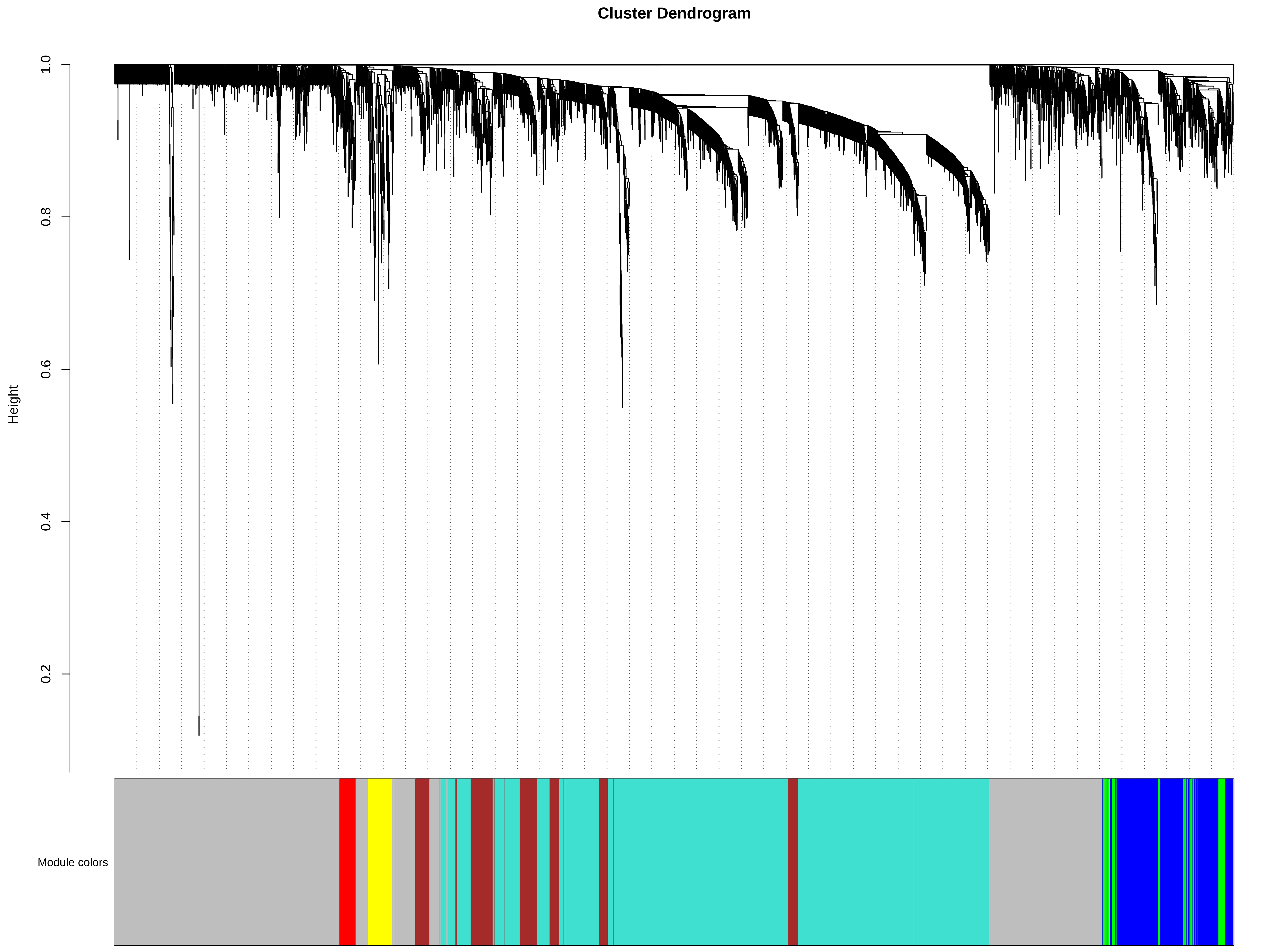

Cluster Dendrogram
Height
1.0
0.8
0.6
0.4
0.2
Module colors
