# Supplementary material for: Integrated bioinformatics and machine learning identify early diagnostic biomarkers for MAFLD with comorbid psoriasis
Source: Front Immunol. 2026 Apr 15;17:1699470. doi: 10.3389/fimmu.2026.1699470 (PMC13125174; doi:10.3389/fimmu.2026.1699470)
Supplement: Supplementary file 1 [file DataSheet1.zip › Supplementary material/code.zip!/code/code/WGCNA/5.gene modules and phenotypes/step5_gene-Module-trait-significance.pdf]

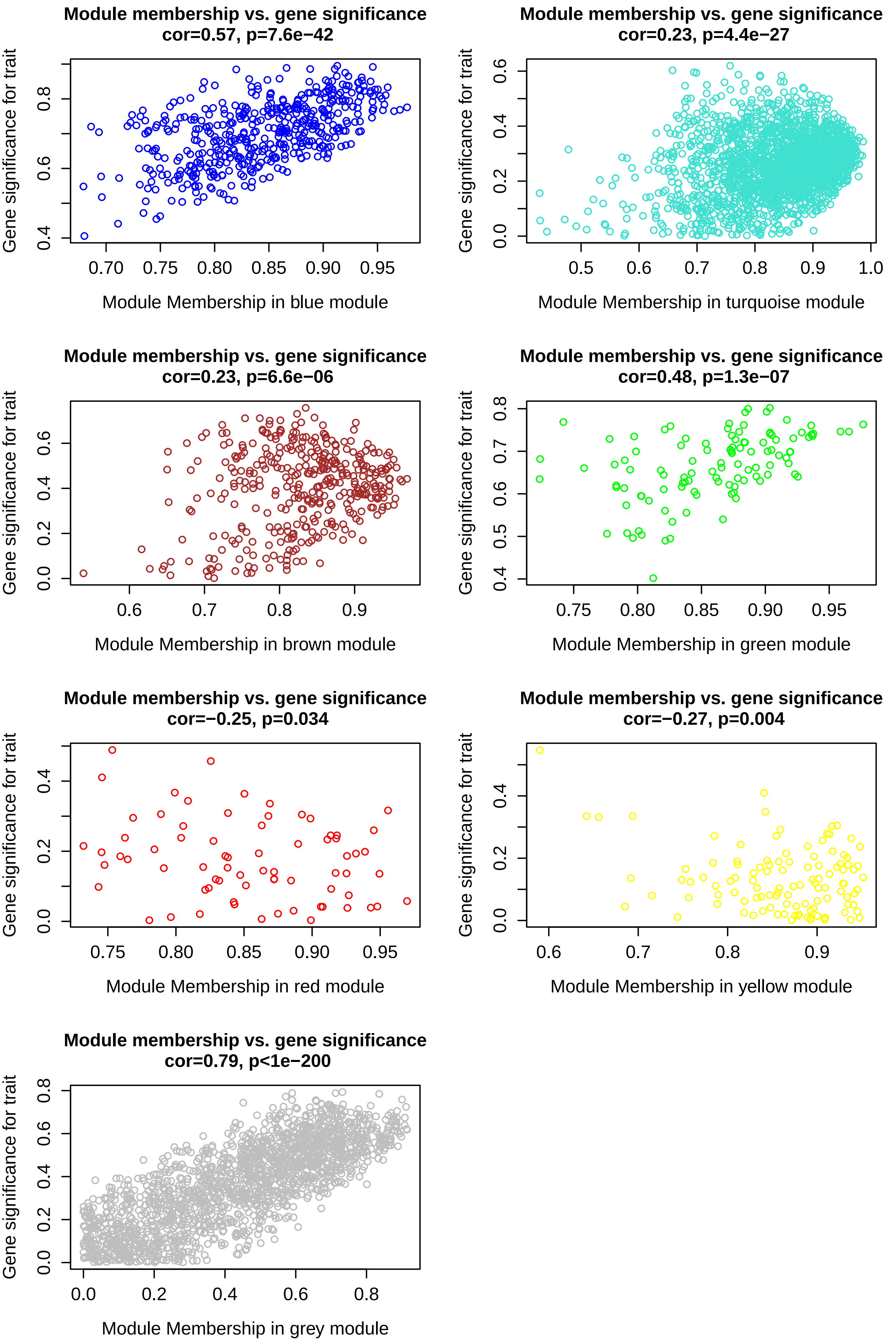
Module membership vs. gene significance
cor=0.57, p=7.6e−42
Gene significance for trait
Module Membership in blue module
Module membership vs. gene significance
cor=0.23, p=4.4e−27
Gene significance for trait
Module Membership in turquoise module
Module membership vs. gene significance
cor=0.23, p=6.6e−06
Gene significance for trait
Module Membership in brown module
Module membership vs. gene significance
cor=0.48, p=1.3e−07
Gene significance for trait
Module Membership in green module
Module membership vs. gene significance
cor=−0.25, p=0.034
Gene significance for trait
Module Membership in red module
Module membership vs. gene significance
cor=−0.27, p=0.004
Gene significance for trait
Module Membership in yellow module
Module membership vs. gene significance
cor=0.79, p<1e−200
Gene significance for trait
Module Membership in grey module
