# Supplementary material for: Integrated bioinformatics and machine learning identify early diagnostic biomarkers for MAFLD with comorbid psoriasis
Source: Front Immunol. 2026 Apr 15;17:1699470. doi: 10.3389/fimmu.2026.1699470 (PMC13125174; doi:10.3389/fimmu.2026.1699470)
Supplement: Supplementary file 1 [file DataSheet1.zip › Supplementary material/code.zip!/code/code/WGCNA/5.gene modules and phenotypes/step5_gene-Module-trait-significance_interested.pdf]

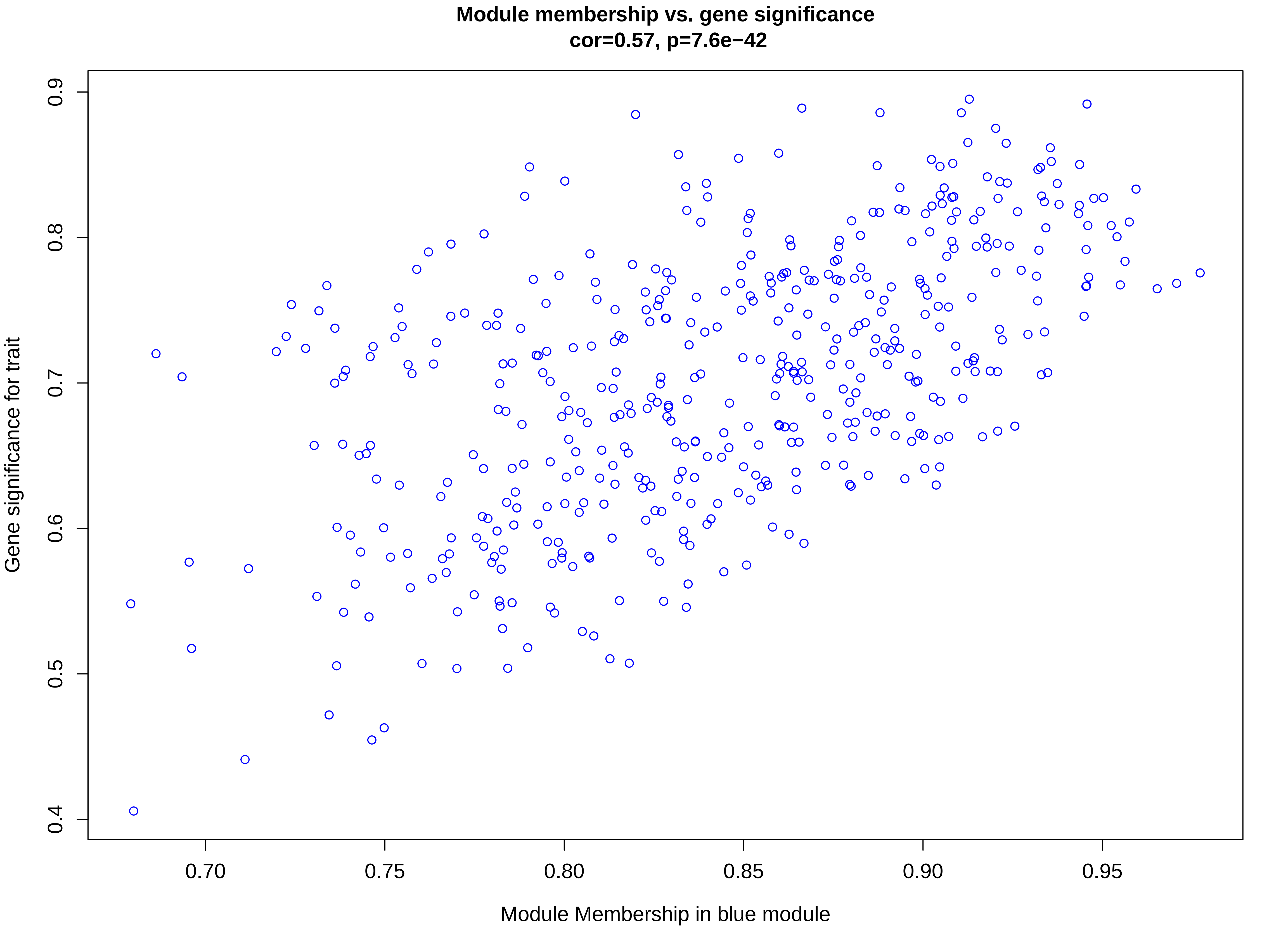
Module membership vs. gene significance
cor=0.57, p=7.6e−42
Gene significance for trait
0.4
0.5
0.6
0.7
0.8
0.9
0.70
0.75
0.80
0.85
0.90
0.95
Module Membership in blue module
