# Supplementary material for: Integrated bioinformatics and machine learning identify early diagnostic biomarkers for MAFLD with comorbid psoriasis
Source: Front Immunol. 2026 Apr 15;17:1699470. doi: 10.3389/fimmu.2026.1699470 (PMC13125174; doi:10.3389/fimmu.2026.1699470)
Supplement: Supplementary file 1 [file DataSheet1.zip › Supplementary material/code.zip!/code/code/WGCNA/5.gene modules and phenotypes/step5_Module-trait-relationship_boxplot_interested.pdf]

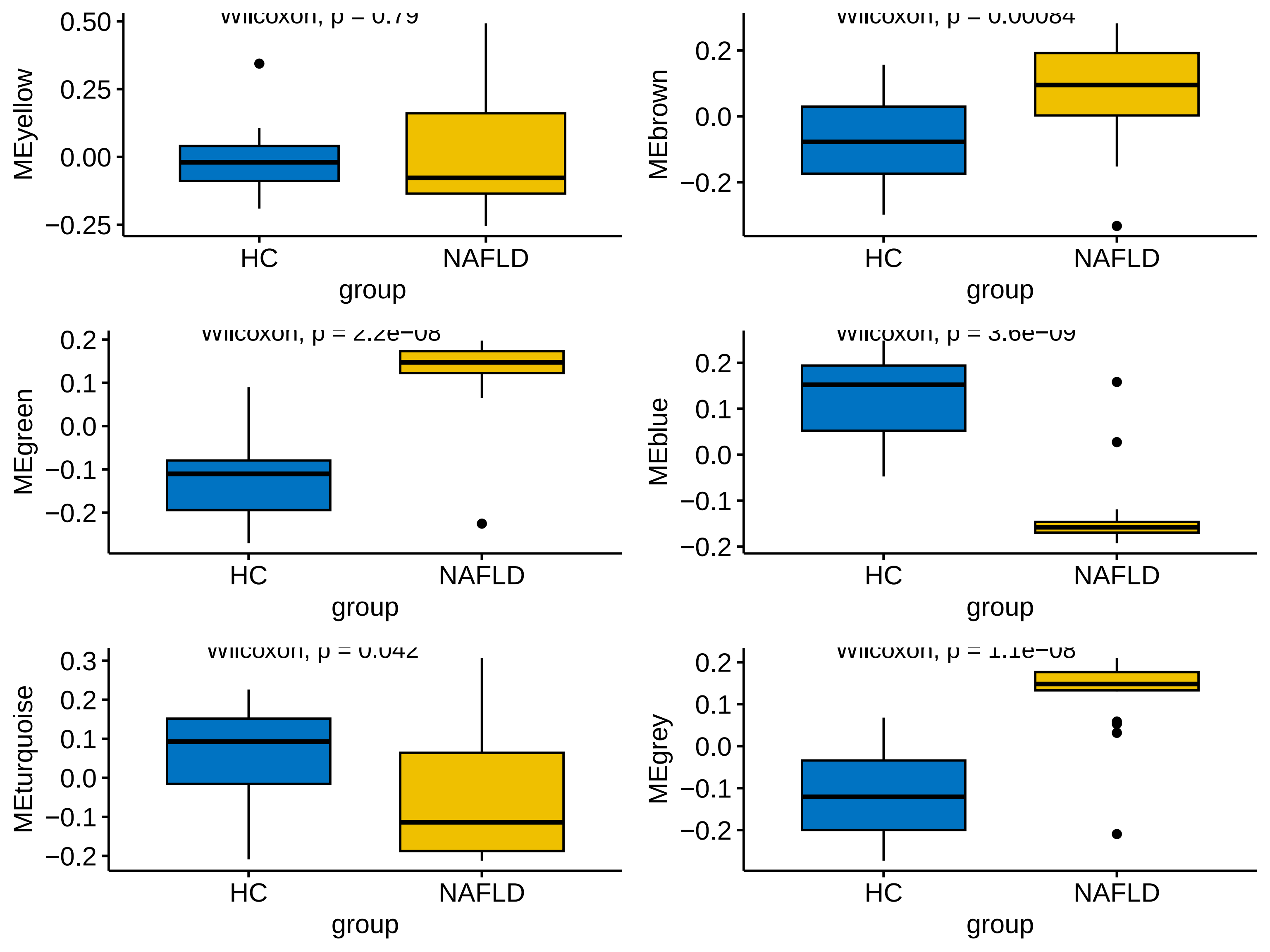
Wilcoxon, p = 0.79
MEyellow
0.50
0.25
0.00
−0.25
HC
NAFLD
group
Wilcoxon, p = 0.00084
MEbrown
0.2
0.0
−0.2
HC
NAFLD
group
Wilcoxon, p = 2.2e−08
MEgreen
0.2
0.1
0.0
−0.1
−0.2
HC
NAFLD
group
Wilcoxon, p = 3.6e−09
MEblue
0.2
0.1
0.0
−0.1
−0.2
HC
NAFLD
group
Wilcoxon, p = 0.042
MEturquoise
0.3
0.2
0.1
0.0
−0.1
−0.2
HC
NAFLD
group
Wilcoxon, p = 1.1e−08
MEgrey
0.2
0.1
0.0
−0.1
−0.2
HC
NAFLD
group
