# Supplementary material for: Integrated bioinformatics and machine learning identify early diagnostic biomarkers for MAFLD with comorbid psoriasis
Source: Front Immunol. 2026 Apr 15;17:1699470. doi: 10.3389/fimmu.2026.1699470 (PMC13125174; doi:10.3389/fimmu.2026.1699470)
Supplement: Supplementary file 1 [file DataSheet1.zip › Supplementary material/code.zip!/code/code/WGCNA/5.gene modules and phenotypes/step5_Module-trait-relationship_heatmap.pdf]

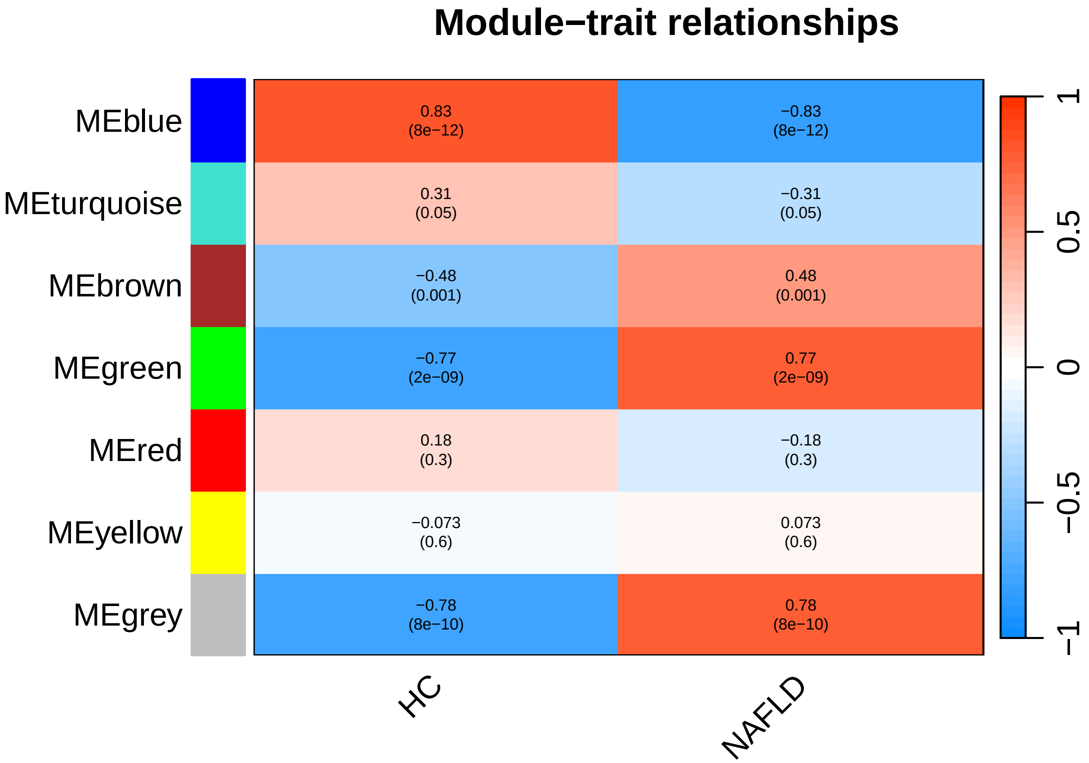

Module−trait relationships
MEblue
MEturquoise
MEbrown
MEgreen
MEred
MEyellow
MEgrey
0.83 (8e−12)
−0.83 (8e−12)
0.31 (0.05)
−0.31 (0.05)
−0.48 (0.001)
0.48 (0.001)
−0.77 (2e−09)
0.77 (2e−09)
0.18 (0.3)
−0.18 (0.3)
−0.073 (0.6)
0.073 (0.6)
−0.78 (8e−10)
0.78 (8e−10)
HC
NAFLD
1
0.5
0
−0.5
−1
